# Implementation of Poliovirus Containment in Poliovirus Designated Facilities—United States, 2017–2024

**DOI:** 10.3390/pathogens14121250

**Published:** 2025-12-06

**Authors:** Christy Ottendorfer, Patrick Vander Kelen, Cecelia A. Sanders, Emily Watson, Suganthi Suppiah, William Hoffman, Colby Sinclair, Abena Marfo, Lia Haynes Smith

**Affiliations:** 1 Centers for Disease Control and Prevention, Office of Readiness and Response, U.S. National Authority for Containment of Poliovirus, Atlanta, GA 30329, USA; 2 Oak Ridge Institute for Science and Education, Oak Ridge, TN 37831, USA; 3 Applied Science, Research & Technology, Inc., Smyrna, GA 30080, USA; 4 Hubert Department of Global Health, Rollins School of Public Health, Emory University, Atlanta, GA 30322, USA

**Keywords:** poliovirus, biorisk management system, laboratory containment, ISO 35001

## Abstract

As the world approaches wild poliovirus eradication, effective containment measures are essential to minimize the risk of a facility-associated reintroduction into a polio-free community. Between 2017 and 2024, the United States established a national authority for containment (NAC) of poliovirus to maintain a national inventory of poliovirus materials and designate facilities to retain polioviruses. Countries with designated facilities are expected to maintain primary, secondary, and tertiary safeguards for facility containment certification. Primary safeguard requirements are assessed through audits following an ISO risk-based process. Standardized data collection and reporting tools were developed in Microsoft Access, and data were analyzed using SAS^®^ 9.4 and R software (version 4.4.3). The NAC conducted 16 audits in three categories: gap assessment (n = 10), Stage 1 audit (n = 3), and Stage 2 audit (n = 3). The NAC found that conformance to the containment standard improved among audit categories (χ^2^ = 94.6, 2 df, *p*-value < 0.0001). In 2024, five audits were conducted according to the revised poliovirus containment standard. Notable gaps were identified in system elements associated with risk assessment, clothing and personal protective equipment, accident/incident, and decontamination/inactivation procedures. Despite compliance with secondary and tertiary requirements, several primary containment elements continue to pose a challenge for facilities, resulting in no US-designated facility achieving full certification.

## 1. Introduction

Since the Global Polio Eradication Initiative (GPEI) was founded in 1988, the global incidence of wild poliovirus has declined from 350,000 cases a year in 176 countries to 12 cases in 2 countries in 2023 [1]. Wild poliovirus (WPV) has three serotypes, with type 2 and type 3 declared eradicated in 2015 and 2019, respectively [2]. Wild poliovirus type 1 remains endemic in Afghanistan and Pakistan [1]. Once global efforts to eradicate poliovirus (PV) from the human reservoir/population are achieved, laboratories (e.g., vaccine manufacturers, research laboratories, and biorepositories) will pose a significant risk of reintroducing the virus into communities [3]. For example, one smallpox laboratory acquired infection, and death occurred after global interruption of variola virus transmission [4], a case that reinforced restrictions on the number of laboratories and containment controls for variola virus [5].

Like the smallpox containment program, the global action plan (GAP) for poliovirus containment standard, first published in 1999, requires countries to survey/inventory laboratories holding these materials and recommends high containment requirements to safeguard these materials [6]. In 2015, the third edition of the containment standard was released [7] followed by the April 2016 synchronized global switch to remove the type 2 component from the live-attenuated oral polio vaccine (OPV) [8]. These actions triggered containment for type 2 polioviruses. As a result, the United States (U.S.) established a poliovirus containment program and a national authority for containment (NAC), identified facilities with PV materials through a national survey, designated domestic facilities to continue critical work or storage of polioviruses, and enrolled these designated facilities in a containment certification program as previously reported [9].

The poliovirus containment standard, third edition, is based on the consensus-based specifications of the CEN workshop agreement 15793 [10], later developed into International Organization for Standardization (ISO) 35001:2019 [11], that employs a management systems approach using a plan–do–check–act (PDCA) cycle to identify, monitor, and control laboratory biosafety and biosecurity [7]. In 2022, the revised fourth edition was released after public consultation with full implementation anticipated by 2026 for all three wild poliovirus serotypes (WPV1, WPV2, and WPV3), vaccine-derived poliovirus serotypes (VDPV1, VDPV2, and VDPV3), and type 2 oral polio vaccine (OPV2) [12]. A three-step process was used for containment certification—(1) certificate of participation (CP), (2) interim certificate of containment (ICC), and (3) certificate of containment (CC)—as previously reported [9]. Globally, 22 countries have seventy-eight designated facilities (also known as poliovirus-essential facilities, PEFs), with most PEFs (54/78, 70%) participating in poliovirus containment certification; one facility achieved full containment certification as of April 2025 [13]. This report describes progress for the implementation of poliovirus containment in the United States.

## 2. Materials and Methods

### 2.1. Facilities Retaining Poliovirus Materials

Research, industrial, clinical diagnostic, environmental, commercial biostorage, and other biomedical facilities in all 50 U.S. states and the 5 populated U.S. territories were surveyed to maintain the national poliovirus inventory as previously reported [9]. Inventory information was collected on facilities holding known polioviruses (known as infectious materials) as well as fecal, respiratory secretion, and/or environmental water samples collected in a geographic area and time when wild poliovirus circulated or OPV was in use (known as potentially infectious materials, PIMs). Facilities reporting typed WPV, VDPV, and OPV2 infectious materials, as well as untyped polioviruses, were contacted by the US NAC to confirm PV inventories, review the poliovirus containment standard, and determine if the facility would apply for containment certification or destroy, inactivate, or transfer these materials in accordance with global eradication milestones. Poliovirus nucleic acids were reported in the survey and monitored as part of the national inventory, but poliovirus nucleic acids did not require containment certification if appropriate conditions [12] and validated methods to inactivate poliovirus were implemented [14]. In addition, novel OPV2 (nOPV2) viruses were reported as OPV/Sabin 2 but have been temporarily waived from containment certification for specific strains and work activities [12]. US NAC also requested strain information for facilities that reported OPV type 1 and OPV type 3 infectious materials to validate Sabin classification in the national inventory. Facilities reported inventory ranges for each serotype, with facilities often reporting more than one type of material.

### 2.2. PEF Applications and Certification Goal

Between 2017 and 2023, facilities that retained WPV2/VDPV2, WPV3/VDPV3, and OPV2 infectious materials applied for the containment CP as previously reported [9]. To prepare for the interruption of WPV1 transmission, facilities retaining WPV1/VDPV1 also applied for CP with self-reported preliminary containment conditions during 2023–2024. PEFs communicated a certification goal to US NAC (e.g., CP, ICC, or CC) with anticipated timelines to achieve the containment standard or conclude work and storage of poliovirus for withdrawal from containment certification.

Prior to CP expiry, PEFs retaining poliovirus types 2 and 3 submitted ICC/CC applications in 2022, consistent with third-edition global deadlines [15]. These applications contained a brief update on the designated facility’s organization, work types, poliovirus inventory, and staffing information since the initial CP application. Applications were reviewed by the US NAC, and audits were scheduled to verify conformance to the poliovirus containment standard.

Facilities that retained OPV/Sabin 1, OPV/Sabin 3, and WPV/VDPV potentially infectious materials were considered non-PEFs and were not designated or enrolled in containment certification during the reporting period. These facilities may become designated facilities in the future as polio eradication milestones are realized, and routine immunization programs discontinue use of OPV.

### 2.3. Training

Auditors completed courses for ISO 9001 [16], ISO 17021 [17], ISO 19011 [18], ISO 35001 [11], and ISO 45001 [19]. Auditors also participated in GPEI-sponsored advanced auditor training to align national laboratory audit and auditor competency processes for poliovirus containment certification of biorisk management systems. The US NAC contracted a technical expert to consult on root cause analysis/corrective action and preventative action plans (RCA/CAPA), risk assessment, and documentation review for auditors. Additionally, the US NAC developed auditor training on the containment standard and interpretation of its requirements, verified by written examination and auditor competency assessments during witnessed audits.

The US NAC provided training webinars and introductory site visits to review containment elements with PEF partners (e.g., top management, laboratory, biosafety, occupational health, facilities, and security personnel). A GPEI-sponsored series of trainings for PEFs was piloted in the United States, including a one-week instructor-led face-to-face course that reviewed the standard in 2019 [2]. Four additional instructor-led remote courses (topics included containment refresher, document management, risk assessment, and RCA/CAPA) focused on implementation in 2023. Remote courses were also provided in 2024 for the standard’s fourth edition.

### 2.4. Poliovirus Containment Standard—Elements

The containment standard is grouped into several technical categories (also known as elements) that have performance-based and prescriptive clauses (e.g., physical laboratory features and worker immunizations) for facility biorisk management systems [7,20]. The standard’s number of elements and clauses was updated between the third (16 elements, 139 clauses) and fourth editions (14 elements, 158 clauses) to consolidate 4 clauses and add 23 new clauses in July 2022 (Supplemental Figure S1). The fourth edition also integrated risk-based approaches for containment controls to update prescriptive requirements from the third edition (e.g., security controls, worker poliovirus vaccination and titer requirements, and physical laboratory features such as a walk-through exit shower). PEF conformance to elements and clauses was assessed by auditors during site visits.

### 2.5. US Poliovirus Containment Policy and Guidance

Many element clauses in the containment standard contain guidance to PEFs on appropriate containment controls. The GAP guidance may be considered by national regulatory authorities as part of certification audits to determine the PEF’s conformance to a clause unless a suitable equivalent is accepted. The US NAC drafted poliovirus containment standard policies applicable to US laboratories consistent with the country’s maintenance of high poliovirus immunization coverage and sanitation standards. US NAC policies translate the containment standard and applicable regulations into meaningful requirements and guidance to promote effective containment and mitigation efforts. While some countries have codified the poliovirus containment standard in national legislation, the US NAC employs a collaborative agreement with designated facilities for containment implementation.

The US NAC policy-making process began with an evaluation of the poliovirus containment standard and applicable U.S. and international regulations and guidelines. Policies were written with the US NAC’s interpretation of these source materials, followed by independent review by both internal and external subject matter experts prior to publication. PEFs were included in the policy and guidance development process to provide transparency and to collect their opinions on draft documents. The US NAC also released interim guidance for US PEFs with additional information to prepare them for certification audits.

US NAC policies are subject to modification and updates based on external circumstances, including new source material such as updated standards or guidelines, changes in law, or changes in eradication status. Revisions are made following the same process as for initial publication, when necessary. PEF conformance to US NAC policies was assessed by auditors during site visits, with policy-specific citations reported in associated audit findings when applicable.

### 2.6. Poliovirus Containment Certification Audits

**Figure 1:**
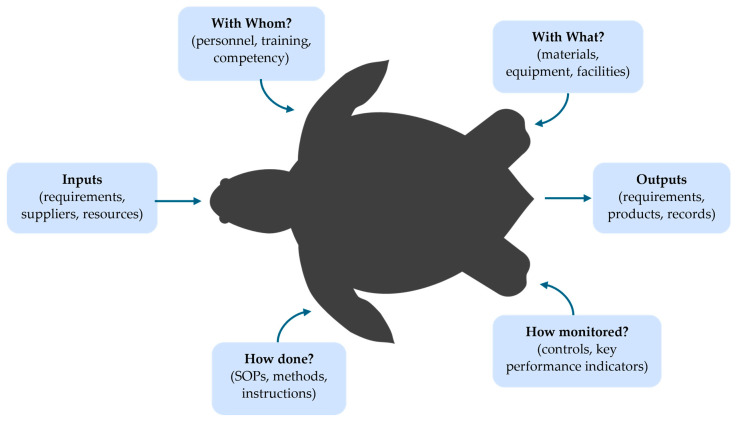
Process auditing “turtle diagram” tool used to audit elements of the poliovirus containment standard. The tool uses a comprehensive overview of a process by listing the necessary documents, related processes, process owner and other support personnel, the equipment and resources used, and process controls and criteria used to meet objectives. The information in each respective box was completed by auditors to assess PDCA principles for each process element. SOPs—standard operating procedures.

The US NAC established an audit team as previously reported [9]. CDC technical experts in mechanical engineering, veterinary medicine, and wastewater conveyance and treatment systems supported certification audits. For the U.S. national laboratory, an external lead auditor conducted the certification audits to mitigate a potential conflict of interest with US NAC auditors employed by the same institution. The US NAC audit processes align with ISO 19011:2018 [18] for auditing management systems. Audits consisted of laboratory and support area tours, personnel interviews, and document review. Audits used a process approach with turtle diagram tools to visualize and guide interviews about each element (Figure 1) [16]. Audit days were calculated as the number of remote or onsite days multiplied by the number of assigned auditors, consistent with ISO 17021:2015 [17] and International Accreditation Forum (IAF) mandatory document (MD) high-complexity business sector for occupational health and safety management systems guidance (IAF MD5:2023) [21]; associated audit planning and reporting activities were excluded from the analysis.

Two pilot audits were used to develop standard assessment criteria in 2017. Gap assessments were conducted between 2019 and 2023, with audit scope determined by PEFs (i.e., partial or comprehensive for all elements). These gap assessments were structured reviews to assess PEFs’ readiness for a certification audit. Findings were communicated in writing following these assessments; however, facilities were not required to provide a formal response to identified findings. Between 2022 and 2023, certification audits were full scope to 16 elements (third edition), and audits were completed in two stages aligned with ISO 17021:2015 [17]. In January 2024, the US NAC retired the third edition and transitioned containment certification audits to incorporate the 14 elements in the fourth edition. Stage 1 audits consisted of document review and short interview sessions conducted via remote video conference. Areas of concern identified during the Stage 1 audit were reported to the PEF to act on prior to the onsite audit. Stage 2 audits were conducted onsite to verify containment implementation, with findings reported and nonconformities categorized as major or minor as described in ISO 19011 [18]. All nonconformity (NC) findings required a formal response from the facility with root cause analysis and a corrective action plan. Surveillance audits were planned within one year of certificate issuance for continued monitoring.

### 2.7. PEF Certification

PEFs holding a CP that completed Stage 1 and Stage 2 audits could receive containment certification. Three safeguards were assessed for certification. The main safeguard focused on whether the PEF met the poliovirus containment standard, as confirmed by the Stage 2 audit, which included the facility’s application, audit finding report, risk assessments, and RCA/CAPA for identified nonconformances. The secondary community safeguard required high (>90%, a minimum of two doses of IPV, i.e., inactivated polio vaccine) population immunity achieved through the national routine childhood polio immunization policy [22]. National and local annual vaccination coverage estimates for 3 or more doses of polio vaccine for children aged 19–35 months who resided in the United States and in specified counties within a 100 km radius of each laboratory address were determined [23]. The tertiary environmental safeguard required PEF placement in areas with demonstrated low poliovirus R0 such as facility connection to a private or public wastewater treatment system that achieves a minimum of secondary treatment of effluents [7,12]. The 1972 Clean Water Act (33 U.S.C. §1251) established requirements for a national pollutant discharge elimination system (NPDES) permit program in the United States [24,25]. A comprehensive facility application package contained data on each safeguard for review by the US NAC Director and a second CDC reviewer independent of the CDC/US NAC audit program and CDC/Polio and Picornavirus Branch. Consistent with ISO 17021 [17], PEF applications were processed and endorsed, and time-bound certificates were issued as previously reported [9].

### 2.8. Information Collection

CDC determined that the information collection activities conducted under the project are exempt from the requirements of the Paperwork Reduction Act (PRA) as they fall under the activities authorized under the National Childhood Vaccine Injury Act (NCVIA) in section 2102(a)(6)–(a)(7) of the Public Health Service Act (42 USC 300aa-2(a)(6)–(a)(7). This activity was reviewed by the CDC and was conducted in accordance with applicable federal law and CDC policy (see, e.g., 45 C.F.R. part 46, 21 C.F.R. part 56; 42 U.S.C. §241(d); 5 U.S.C. §552a; 44 U.S.C. §3501 et seq).

### 2.9. Data Analysis

The US National Inventory for Poliovirus Containment survey dataset was queried for records collected between 2015 and 2024, with 2015–2016 survey records combined with the year 2017, consistent with when the US poliovirus containment program was established. Data were extracted to determine US facilities that reported poliovirus infectious material subject to containment certification. Audit data were validated from the 16 audit events (third edition) and 5 audit events (fourth edition) that occurred. To evaluate the effectiveness of the multi-stage audit process for element conformance (third edition), a Chi-Square Test of Independence was executed in SAS^®^ 9.4 software (SAS Institute Inc., Cary, NC, USA) [26]. Descriptive statistical analyses were conducted in Microsoft 365 Excel version 2102 to determine the proportion of facilities by state with a reduction in containment serotypes held and the proportion of element clauses where nonconformance was found for the three audit categories (gap assessment, ICC Stage 1, and ICC Stage 2), as well as to compare the pass rates in element clauses for certification audits to analyze trends and identify elements for additional clarification or technical guidance, as well as continual improvement of program implementation. In addition, the frequency of U.S. NAC policy areas cited in the third-edition findings was also analyzed based on policy effective dates between 2018 and 2023. To evaluate facility-specific element improvement during audits, the Mann–Whitney U test was conducted in R Studio with R4.4.3 software [27] to test for statistical significance in the number of nonconformance element clauses between ICC Stage 1 and ICC Stage 2 groups due to the small facility sample size (n = 3) and the third-edition nonconformance data not being normally distributed. The U.S. map was created in ArcGIS version 10.7.1. to geographically visualize the reduction in facilities containing poliovirus infectious materials from 2017 to 2024.

## 3. Results

### 3.1. Facilities Retaining Poliovirus Materials

**Figure 2:**
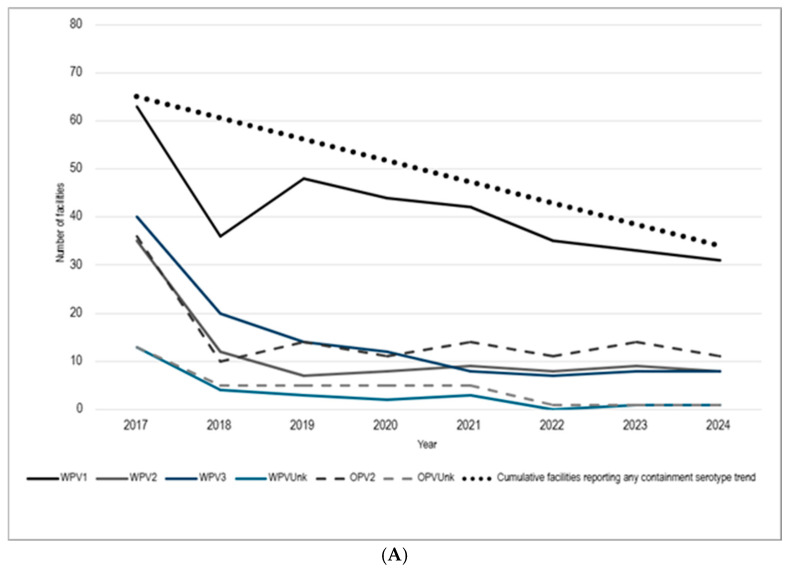

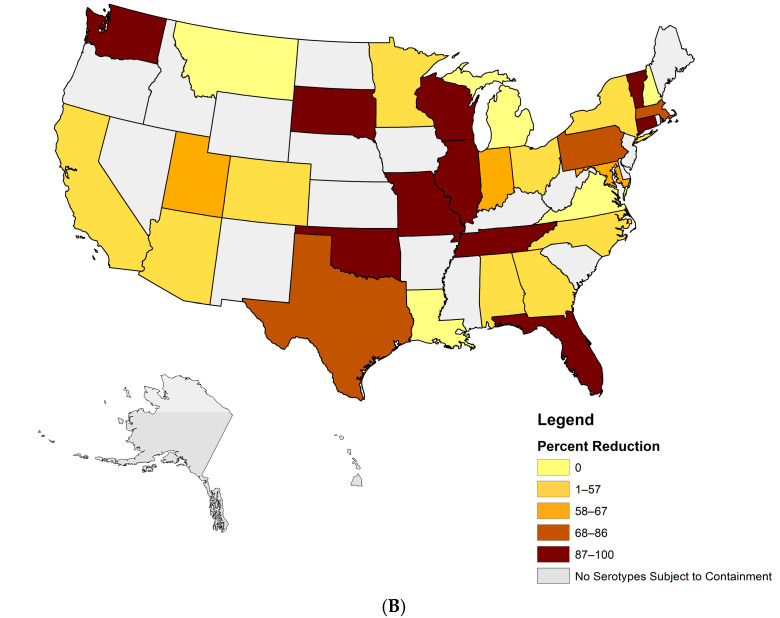
Trends in poliovirus infectious materials reported by serotype, United States, 2017–2024. (**A**) Number of facilities reported in the national survey by serotype (including PV nucleic acids and nOPV2 infectious materials) subject to poliovirus containment standard between 2017 and 2024. Some facilities reported having more than one serotype including WPV1—wild and vaccine-derived poliovirus type 1; WPV2—wild and vaccine-derived poliovirus type 2; WPV3—wild and vaccine-derived poliovirus type 3; WPVunk—untyped wild and vaccine-derived poliovirus; OPV2—oral polio vaccine Sabin type 2; and OPVunk—untyped oral polio vaccine infectious materials. (**B**) Map of national survey facility respondents by state with containment of poliovirus infectious materials (2017–2023) compared to facility holdings in 2024. Facilities with reported OPV/Sabin 1 and OPV/Sabin 3 infectious materials and potentially infectious materials were excluded from the analysis.

A cumulative total of 100 different facilities reported poliovirus infectious materials subject to containment certification in the national survey (Figure 2). Containment implementation resulted in large reductions in facilities retaining poliovirus infectious materials by serotype, including WPV2/VDPV2 (35 to 8, 77.1% reduction), OPV2 (36 to 11, 69.4%), WPV3/VDPV3 (40 to 8, 80%), WPV1/VDPV1 (63 to 31, 50.8%), and both WPV and OPV untyped materials (13 to 1, 92.3%). Some facilities reported more than one serotype, and ten facilities were excluded from containment certification based on material characteristics (six facilities reported PV nucleic acids only, while four facilities reported nOPV2). Facility reductions were also stratified by state for states with facilities reporting a containment serotype. Of these 30 states, 10 states had a 100% reduction in facilities holding containment strains during the reporting period (Figure 2). Validation of the national survey data revealed that 5 out of 14 facilities (35.7%) misclassified WPV1 strains (e.g., CHAT and Mahoney) as OPV/Sabin 1. In addition, inventory corrections were requested by facilities to remove errors in serotype and materials reported.

### 3.2. PEF Applications and Certification Goal

**Figure 3:**
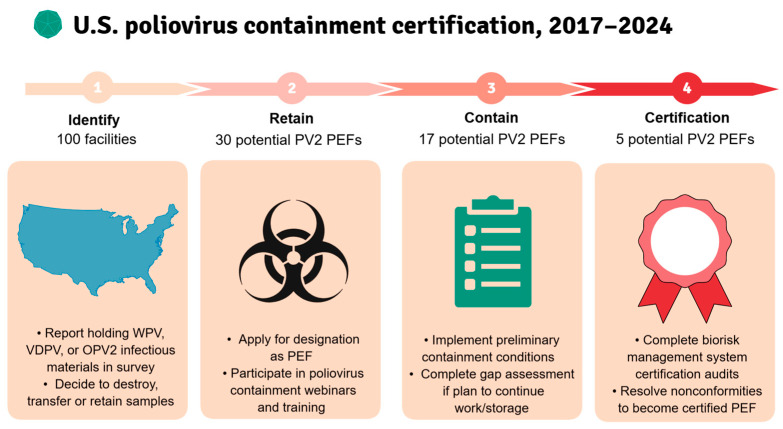
A multi-step process was used to identify, designate, and certify potential PEFs in the United States. In 2017, the US NAC initiated containment for facilities reporting poliovirus type 2 materials in the national survey (potential PV2 PEFs), followed by containment of retained WPV3/VDPV3 infectious materials at these facilities in March 2021. Poliovirus containment certification has resulted in a continued decrease in the number of potential PV2 PEFs, with only 5 potential PV2 PEFs progressing to interim certification.

From 2017 to 2024, many US facilities reported holding poliovirus infectious materials from all serotypes subject to containment certification. Initially, the US NAC focused on poliovirus type 2 and WPV3/VDPV3 infectious materials with 30 potential PEFs designated as previously reported [9]. Of these, 17 potential PEFs had a CP endorsed based on preliminary containment conditions implemented. In 2022, five out of ten CP-holding designated facilities submitted ICC applications to continue retaining PV2 and WPV3/VDPV3 materials (Figure 3). Two PEFs destroyed or inactivated materials and withdrew from containment certification (after completing gap assessments), and one PEF was lost to follow-up after the US NAC’s CP endorsement. During 2023–2024, 20 out of 41 WPV1 facilities submitted CP applications to retain WPV1/VDPV1 materials, including 2 PEFs that destroyed or transferred PV2 and WPV3/VDPV3 materials and re-applied as WPV1 PEFs. Five PV2/WPV3/VDPV3 facilities also held WPV1 materials, and the remaining sixteen facilities destroyed, transferred, or inactivated WPV1 materials.

Characteristics of US PEFs engaged in the containment certification program are shown in Table 1. Four PEFs retaining PV2 and WPV3/VDPV3 relocated or transitioned work with these serotypes into laboratories designed to A/BSL-3 standards described in the Biosafety in Microbiological and Biomedical Laboratories (BMBL) 6th edition [28], including one facility that designed and constructed a new poliovirus containment area with physical laboratory features as outlined in the poliovirus containment standard [12]. In contrast, most WPV1 PEFs continued work in A/BSL-2 laboratories and planned to withdraw during the CP period. As of 31 December 2024, two WPV1 PEFs withdrew from containment certification and destroyed materials.

### 3.3. Training

The GPEI-sponsored training courses for auditors and advanced auditor training supported the US NAC’s development of appropriate audit guidance and tools for biorisk management system audits and auditor calibration activities. However, GPEI-sponsored training courses were primarily organized by region based on geography on an annual or biennial cycle, with limited seats available, and experienced disruptions during the COVID-19 pandemic. These limitations led the US NAC to develop an auditor training and competency program on the poliovirus containment standard to ensure appropriate audit planning and staffing capacity. US PEFs reported positive feedback to the US NAC on training courses and requested continued support of training initiatives on biorisk management systems for poliovirus containment.

### 3.4. Poliovirus Containment Standard—Elements and Audits

Seven potential U.S. PV2 PEFs completed gap assessments to the poliovirus containment standard, third edition. Five facilities applied for certification, and ICC audits were scheduled. Of the five, three facilities completed ICC audits to GAPIII. Two facilities completed ICC audits to GAPIV (Supplemental Figure S2). No containment audits were conducted at potential WPV1/VDPV1 PEFs.

#### 3.4.1. GAPIII Elements and Audits

**Figure 4:**
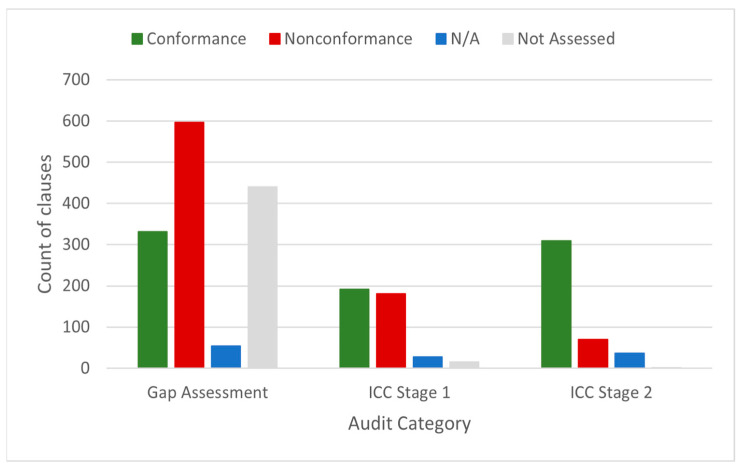
Conformance to poliovirus containment standard (3rd edition) element clauses by audit category. The US NAC conducted gap assessments (n = 10) at 7 PE Fs and paired ICC audits (n = 6) at 3 PEFs, retaining PV2 and WPV3/VDPV3 infectious materials. The number of third-edition clauses was constant during the reporting period, but the gap assessment category dataset had over three times the number of audits compared to the ICC stages. N/A—not applicable.

A total of 16 audits at 7 PEFs were conducted to determine conformity to the poliovirus containment standard (third edition) during 2017–2023. The median duration of audits was 9 days (min = 4, max = 20). The number of PEFs decreased by two after the gap assessment category, with five designated facilities holding PV2 and WPV3/VDPV3 materials seeking certificates beyond the CP. Between 2022 and 2023, three out of five PEFs completed Stage 1 and Stage 2 audits for interim containment certification. Conformance to third-edition element clauses significantly improved among audit categories (χ^2^ = 94.6, 2 df, *p*-value < 0.0001) and between the ICC Stage 1 and ICC Stage 2 audit categories (U = 193.5, z = 2.46, *p* = 0.01386) (Figure 4).

Nonconformance varied by element when assessed by the US NAC (Table 2). For example, a high proportion (>60%) of nonconformance was observed for 10 out of 16 elements during gap assessments, including systems used for personnel, human factors, accident/incident, equipment and maintenance, decontamination, and transfer controls. Despite significant improvement, ICC Stage 2 containment certification audits resulted in several nonconformance clauses grouped into a mean of 6 nonconformities (NCs; range: 0–15) in the primary facility safeguard. Most PEFs initially reported concerns with achieving the prescriptive physical facility element, but these results suggest other elements were also complex or challenging to fully implement.

#### 3.4.2. GAPIV Elements and Audits

A total of five audits at three PEFs were conducted to determine conformity to the poliovirus containment standard (fourth edition) in 2024. A median of 9 audit days (min = 9; max = 12) was observed. Although two elements were combined into fourteen in the fourth edition, the reduction in the number of elements was offset by an increase of 23 new clauses distributed in nine elements compared to the third edition (Table 3). Nonconformance results varied by element, with a high proportion (≥60%) found for five elements in the fourth edition. These fourth-edition containment certification audits also resulted in several clauses grouped into a mean of 11 NC findings (range: 6–17) in the primary facility safeguard, an increase compared to the third edition. Despite the fourth-edition revision to a risk-based approach, new nonconformities were identified for two PEFs compared to their prior third-edition gap assessments. Further, one PEF had zero nonconformities to the standard’s third edition but six nonconformities to the fourth edition, resulting in corrective actions needed prior to achieving full certification.

### 3.5. US Poliovirus Containment Policy and Guidance

**Figure 5:**
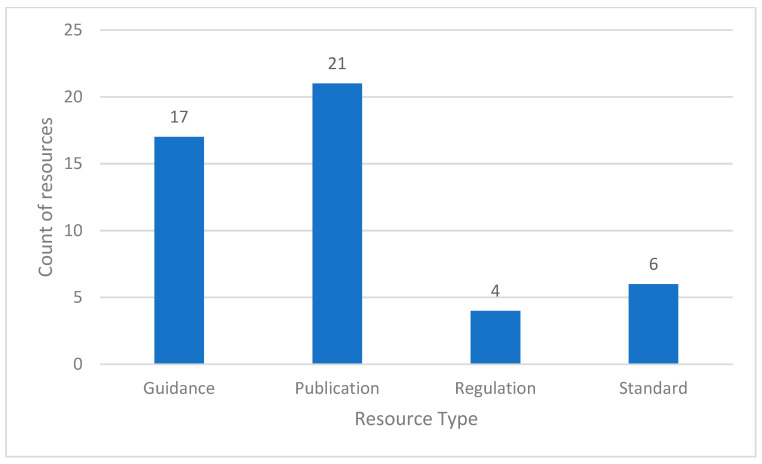
Resource types used in the US NAC policy area development. US NAC policies included a small number of U.S. and international regulations but relied on publications to support poliovirus containment implementation in the United States.

Ten poliovirus containment policy areas were developed, published, and analyzed to address the US NAC’s oversight of the containment standard (third edition). Policy inputs were categorized by source type, including regulations, standards, guidance, and publications (Figure 5). The US NAC relied on publications (e.g., the scientific peer-reviewed literature, book chapters, and technical documents) to make data-driven recommendations, with 44% (21) of the total number of references used for policy development. Guidance documents were the second largest input into policy areas (e.g., US government and intergovernmental resources), with 35% (17) of total references, followed by standards (e.g., ISO and National Institutes of Health), consisting of 13% (6) of the total references. Lastly, US regulations (e.g., CDC Import Permit Program (IPP), Department of Transportation (DOT), and Occupational Safety and Health Administration (OSHA)) were the remaining policy area inputs with 8% (4) of the total references.

To support facility preparations for certification audits, three interim guidance documents were released to US PEFs to support reporting information associated with identified NCs. Example templates for major NC risk assessment, audit finding root cause analysis, and corrective action plans were developed and reviewed with designated facilities. The interim guidance provided a standard format and recommendations on how to respond to audit reports, resulting in improved application information.

Facility conformance to US NAC policy areas was evaluated in the 16 audits (third edition) conducted. The US NAC reported a total of 582 findings, where 62 findings were associated with one or more of the ten US NAC policy areas (n = 70). Policy areas for security (including access records) and biorisk management systems/risk assessment were most often cited (21.4%). In contrast, the PPE and hand hygiene practices, shared use of space, and transfer policies were each cited less than five times, totaling less than 13% of all policy area citations. When adjusted by time in effect, emergency response and exposure management plans (0.67) and occupational health (0.42) policy areas had the highest citation rates (Table 4).

### 3.6. PEF Certification Decision

No PEF was fully certified to the third edition due to the release of the standard’s fourth edition during the reporting period. PEFs continued to implement corrective actions to resolve NC findings and come into full compliance with the primary facility safeguard. National and local designated facility jurisdiction immunization coverage estimates met or exceeded 90% for two doses of inactivated poliovirus vaccine for the secondary community safeguard. Wastewater treatment utilities connected to US PEFs had NPDES permits issued by state regulatory authorities. As of March 2025, three US PEFs were issued an interim certificate of containment, one PEF completed the ICC application/audit and received US NAC endorsement, and one designated facility was prepared to withdraw from certification following the Stage 2 audit. An average of 19 months was found for the issuance of an interim certificate from the facility ICC application date.

## 4. Discussion

Since the GPEI launched in 1988, poliovirus has been eliminated from most countries, with two of three wild poliovirus serotypes declared eradicated worldwide [1]. As public health efforts intensify to interrupt transmission of WPV1 and control VDPV outbreaks [29,30,31,32,33], the United States has committed to safeguarding the eradication initiative through the identification of domestic facilities holding poliovirus materials (national survey) and implementation of stringent biocontainment measures [34]. Recent laboratory-acquired infections (LAIs) reported in poliovirus facilities in other countries suggest that enhanced containment practices are critical to mitigate facility-associated risk of poliovirus reintroduction into communities [35,36]. Conformance to the poliovirus containment standard is not compulsory for eradicated WPV serotypes in the United States, whereas variola virus (smallpox containment) is subject to the select agent regulations [37]. As a result, the US NAC established oversight of a poliovirus laboratory containment certification program aligned to ISO management system standards (ISO17021 [17] and ISO 35001 [11]), national policies, and the global poliovirus containment standard [7,12].

The number of facilities reporting poliovirus infectious materials subject to containment certification has varied over time, as previously documented [9]. The increase in facilities reporting WPV1/VDPV1 in 2019 was attributed to a large survey launch to grant recipients conducted between fall 2018 through 2019. These observed fluctuations over time were consistent with the ongoing evaluation of poliovirus inventory in the US through continuous outreach conducted by the US NAC to increase survey participation. As of 2024, a total of 25 facilities reported infectious materials, excluding nOPV2 and PV nucleic acids, representing an overall reduction of 74%. A moderate percentage (35.7%) of facilities reporting OPV/Sabin 1 materials had non-Sabin strains defined as WPV1 materials in the poliovirus containment standard [12]. Since initial worldwide survey efforts were focused on wild poliovirus infectious and potentially infectious materials, and then OPV/Sabin 2 materials aligned with the 2016 global switch from trivalent to bivalent oral polio vaccine [38,39,40,41,42,43], the US NAC found that inventory of all three poliovirus serotypes, data validation, and collection of strain information were essential to ensure appropriate facility designation and implementation of enhanced containment measures. These findings suggest that countries should verify the strain characteristics of oral polio vaccine (OPV) materials held by facilities. If initial inventories of poliovirus types 1 and 3 have not been finalized, or if additional surveys are planned, countries should consider collecting this information from facilities [34]. As global eradication milestones and post-certification goals are realized, containment controls for remaining OPV/Sabin vaccine serotypes will be expanded in the United States.

The United States has the largest number of PEFs enrolled in containment certification worldwide [13]. Due to the high proportion of NC clauses observed during gap assessments, the US NAC established a phased approach for auditing a new laboratory biorisk management standard for US poliovirus laboratories. Consistent with ISO 17021:2015, initial certification audits were conducted in two stages, with significant improvement in conformance observed among audit categories for US PEFs retaining PV2 and WPV3/VDPV3 materials. This oversight strategy allowed PEFs additional time to develop, implement, and improve site-specific biorisk management systems, resulting in a lower number of Stage 2 audit NC findings that required corrective action. However, some system elements continued to pose challenges for full containment certification of the primary facility safeguard—risk assessment, clothing and personal protective equipment, accident/incident, and decontamination/inactivation—irrespective of the third or fourth edition of the standard audited. Although significant improvement in containment practices was found during this reporting period, some PEFs that could not meet stringent biorisk management system elements opted to conclude work/storage of containment PV serotypes, with destruction and/or transfer of these materials to other designated facilities for collaboration on critical research activities aligned with global action plan strategies [12]. As of April 2025, only one PEF has achieved full certification to the standard worldwide, which suggests that the revised standard maintains a high level of difficulty for implementation [13]. The US NAC plans continued engagement with U.S. PEFs and other national authorities to foster a community of practice for consistent implementation of poliovirus containment to safeguard the polio eradication efforts.

In 2022, WPV1 case numbers were the lowest recorded [44], resulting in a global eradication certification commission announcement that all wild poliovirus serotypes were subject to immediate containment certification [29]. The US NAC found that facilities often transitioned work activities to serotypes not yet subject to containment requirements rather than implement the more stringent biorisk management system, with some PEFs later re-entering containment certification for WPV1 materials. By December 2024, the US NAC successfully enrolled 20 PEFs that retained WPV1 infectious materials, reducing the number of WPV1 facilities by half. However, the US NAC anticipates the progression of WPV1 facilities through the certification process to align with global epidemiological data and eradication milestones. A recent increase in WPV1 cases suggests that interruption of transmission will be delayed [45,46], with WPV1 PEFs moving forward with initial containment conditions as previously reported [9] and preparing to implement biorisk management systems consistent with the poliovirus containment standard.

The United States government does not maintain a national registry of all laboratories; instead, the US NAC relies on a national survey and policies for reporting poliovirus facility inventories and enrollment in containment certification. The US NAC found that policy area development was shaped more by domestic subject matter experts, the scientific literature, and US government guidance than by binding international regulations or standards. US policies helped bridge gaps that standards drafted for a global audience with varying infrastructure, capacity, and capabilities may have available to implement. For example, security and emergency management policy areas contained robust controls not clearly specified or mandated in the containment standard, but they were aligned to local capacity available in the United States, resulting in audit citations. The US NAC anticipates continual improvement of program activities to strengthen domestic oversight consistent with the January 2025 Executive Order 14155, withdrawing the United States from the World Health Organization [47]. Future work is expected to strengthen bilateral relationships with countries on containment practices and improve the poliovirus containment standard based on audit data, consensus-based biocontainment standards, and industry best practices for biorisk management systems. 

This report is the first to describe poliovirus containment audits of laboratory biorisk management systems consistent with ISO 35001 principles and the PDCA cycle for biocontainment in the United States. Although PV2-holding facilities were experienced in BSL-3 operations consistent with national biosafety guidance [28] and/or regulation [37], US PEFs reported alignment of multiple technical areas and work units to the poliovirus containment standard that required additional resources, engagement, and effort for certification to retain infectious PV2 and WPV3/VDPV3 materials. Similarly, a gap analysis conducted at five national reference laboratories in three European countries to CWA 15793 (predecessor of ISO 35001) found that robust containment programs were in place, but identified gaps in biorisk management system development and compliance [48]. While ISO 35001 biorisk management system models have been discussed in the United States [49,50,51], limited adoption has been reported both in the United States [52,53] and worldwide [54,55,56]. Poliovirus-designated facilities continue to pioneer laboratory biorisk management system implementation with certification audits conducted by national regulatory authorities. Future innovation in biosafety programs may consider implementing a systems-based management standard to strengthen and continuously improve the overall performance of containment laboratories to minimize the risks associated with work using consequential pathogens.

Limitations of the report include the following: (1) The poliovirus vaccine is not manufactured in the United States, and thus, USA containment findings may not be representative of PEFs that produce poliovirus vaccines. (2) Audit methods collect a sample of information, resulting in uncertainty that nonconformities were present but not detected by the audit team. (3) Audit teams are composed of different auditors who can introduce variation in the identification of findings. (4) A reduction in PEFs during the steps in the certification process limited a full comparative analysis by facility features. Some containment clauses were not assessed by auditors on each audit event. The small number of PEFs posed limitations for statistical analysis; however, this limitation will persist as countries worldwide continue to reduce the number of facilities containing poliovirus. (5) The U.S. does not have a national laboratory registry; therefore, the national survey data may be incomplete, and certain industries may be undersampled.

## Figures and Tables

**Table 1:** Characteristics of U.S. facilities designated to retain poliovirus by virus type, 2022–2024.

	Virus Type			
PEF Characteristics ^a^	^b^ PV2, WPV3/VDPV3		WPV1/VDPV1	
N = 25	Number (n = 5)	Percent (%)	Number (n = 20)	Percent (%)
*Facility Type*				
Academic	1	20	11	55
Commercial	2	40	7	35
Government	2	40	2	10
*Work Types(s)* ^c^				
Research ^d^	3	60	15	75
Vaccine Production	0	0	0	0
Clinical Trials	2	40	2	10
Animal Model	1	20	4	20
Diagnostics	2	40	1	5
QC Testing ^e^	1	20	6	30
Environmental	-	-	1	5
Storage Only	1	20	0	0
*Laboratory Design* ^f^				
A/BSL-2 ^d^	0	0	14	70
A/BSL-3	4	80	6	30
A/BSL-4	0	0	0	0
Not Applicable (Repository)	1	20	0	0
*Certification Goal*				
Certificate of Participation (CP)	-	-	14	70
Interim Certificate of Containment (ICC)	1	20	0	0
Certificate of Containment (CC)	4	80	6	30

^a^ U.S. PEFs (n = 25) enrolled in poliovirus containment certification. ^b^ PV2—poliovirus type 2 (WPV, VDPV, and/or OPV/Sabin). These facilities also reported WPV1/VDPV1 materials. ^c^ Work type(s)—Facilities reported 2–4 work types in CP applications. An environmental category was added to the application form in 2023. ^d^ Two WPV1/VDPV1 facilities consisted of two principal investigators operating in different laboratory locations. ^e^ QC—quality control. ^f^ A/BSL—animal/biosafety level. Data as of 31 December 2024.

**Table 2:** Heat map of nonconformance in 16 elements (third edition) by audit category.

Element				Audit Category ^1^												
				Count of Nonconformance Clauses Found						Count of Clauses Assessed				Proportion of Nonconformance Clauses Per Element (%)		
				Gap Assessment (n = 10)		ICC Stage 1 (n = 3)		ICC Stage 2 (n = 3)		Gap Assessment (n = 10)		ICC Stage 1 (n = 3)	ICC Stage 2 (n = 3)	Gap Assessment	ICC Stage 1	ICC Stage 2
1 Biorisk management system				217		72		29		345		151	162	62.9	47.7	17.9
2 Risk assessment				46		10		9		62		23	27	74.2	43.5	33.3
3 Inventory				27		5		3		39		15	15	69.2	33.3	20.0
4 General safety				6		2		1		8		3	3	75.0	66.7	33.3
5 Personnel and competency				37		9		2		60		27	27	61.7	33.3	7.4
6 Good microbiological technique				6		3		0		15		6	6	40.0	50.0	0.0
7 Clothing and PPE				7		4		2		16		6	6	43.8	66.7	33.3
8 Human factors				5		1		0		7		3	3	71.4	33.3	0.0
9 Health care				25		6		1		45		18	17	55.6	33.3	5.9
10 Emergency response				32		13		5		56		23	24	57.1	56.5	20.8
11 Accident/incident investigation				5		3		1		7		3	3	71.4	100.0	33.3
12 Physical facility				100		25		9		213		72	72	46.9	34.7	12.5
13 Equipment and maintenance				34		7		3		35		15	15	97.1	46.7	20.0
14 Decontamination				20		7		5		23		12	12	87.0	58.3	41.7
15 Transport				8		2		0		8		3	3	100.0	66.7	0.0
16 Security				22		12		0		43		21	21	51.2	57.1	0.0
Key (%):	0	10	20	30	40	50	60	70	80	90	100					

^1^ The US NAC conducted gap assessments (n = 10) at 7 PEFs and paired ICC audits (n = 6) at 3 PEFs, retaining PV2 and WPV3/VDPV3 infectious materials.

**Table 3:** Nonconformance in 14 elements (fourth edition) in five audits, 2024.

	Element (n) ^a^	Number of Nonconformance Clauses Found ^b^	Number of Clauses Assessed	Proportion of Nonconformance Clauses Per Element (%)
1	Biorisk Management System (1)	81	263	30.8
2	Risk Assessment and Control (1)	37	55	67.3
3	Worker Health Program (0)	16	30	53.3
4	Competence and Training (1)	21	45	46.7
5	Good Microbiological Practice/Procedure (2)	6	16	37.5
6	Clothing and PPE (0)	6	8	75.0
7	Security (0)	10	35	28.6
8	Facility Physical Requirements (7) ^c^	46	152	30.3
9	Equipment and Maintenance (1)	6	10	60.0
10	Poliovirus Inventory and Information (5)	10	40	25.0
11	Waste Management/Decontamination (4)	26	42	61.9
12	Transport Procedures (1)	3	9	33.3
13	Emergency Response/Contingency Plan (0)	16	40	40.0
14	Accident/Incident Investigation (0)	3	5	60.0

^a^ n = number of new clauses in the fourth edition. ^b^ Fourth-edition analysis combined findings identified during ICC Stage 1, ICC Stage 2, and one surveillance audit. ^c^ The fourth edition contains 22 clauses with 1 clause subdivided into 9 subparts for animal facility audits (n = 31). Compared to the third edition, the facility element contains the largest number of new clauses.

**Table 4:** Nonconformance trends in US NAC policy areas cited in sixteen audits of the poliovirus containment standard (third edition) by time in effect, 2018–2023.

US NAC Policy Area	Count Policy Area Citations ^a^ (n = 70)	Proportion Policy Area Citations (%)	Time in Effect (Months)	Rate
Security	15	21.4	60	0.25
Biorisk Management System and Risk Assessment	15	21.4	44	0.34
Emergency Response and Exposure Management Plans	8	11.4	12	0.67
Inactivation	7	10	30	0.23
Inventory	6	8.6	60	0.10
Storage Outside of Containment	5	7.1	60	0.08
Occupational Health	5	7.1	12	0.42
PPE and Hand Hygiene Practices	4	5.7	51	0.08
Shared Use of Space	3	4.3	44	0.07
Transfer	2	2.9	60	0.03

^a^ US policy areas reported in 62 findings. Some findings had one or more policy areas cited for a total of 70. Rate was calculated by number of policy citations divided by months since effective date.

## Data Availability

The datasets generated and analyzed during the current study are not publicly available due to privacy concerns for the poliovirus-essential facilities. Aggregated data is available from the corresponding author upon reasonable request.

## Supplementary Materials

The following supporting information can be downloaded at https://www.mdpi.com/article/10.3390/pathogens14121250/s1: Figure S1: Number of elements and clauses in the poliovirus containment standard by edition; Figure S2: Number of U.S. NAC poliovirus containment audits by facility.

## Abbreviations

The following abbreviations are used in this manuscript:

CAPA	Corrective action preventative action
CP	Certificate of Participation
GAP	Global action plan for poliovirus containment
GPEI	Global Polio Eradication Initiative
ICC	Interim Certificate of Containment
ISO	International Organization for Standardization
NC	Nonconformance
NPDES	National pollutant discharge elimination system
OPV	Oral polio vaccine
PEF	Poliovirus-essential facility
PDCA	Plan–do–check–act
PIM	Potentially infectious material
RCA	Root cause analysis
VDPV	Vaccine-derived poliovirus
WPV	Wild poliovirus
