# Supplementary material for: Implementation of Poliovirus Containment in Poliovirus Designated Facilities—United States, 2017–2024
Source: Pathogens. 2025 Dec 6;14(12):1250. doi: 10.3390/pathogens14121250 (PMC12735635; doi:10.3390/pathogens14121250)
Supplement: Supplementary file 1 [file pathogens-14-01250-s001.zip › pathogens-3926010-supplementary.pdf]

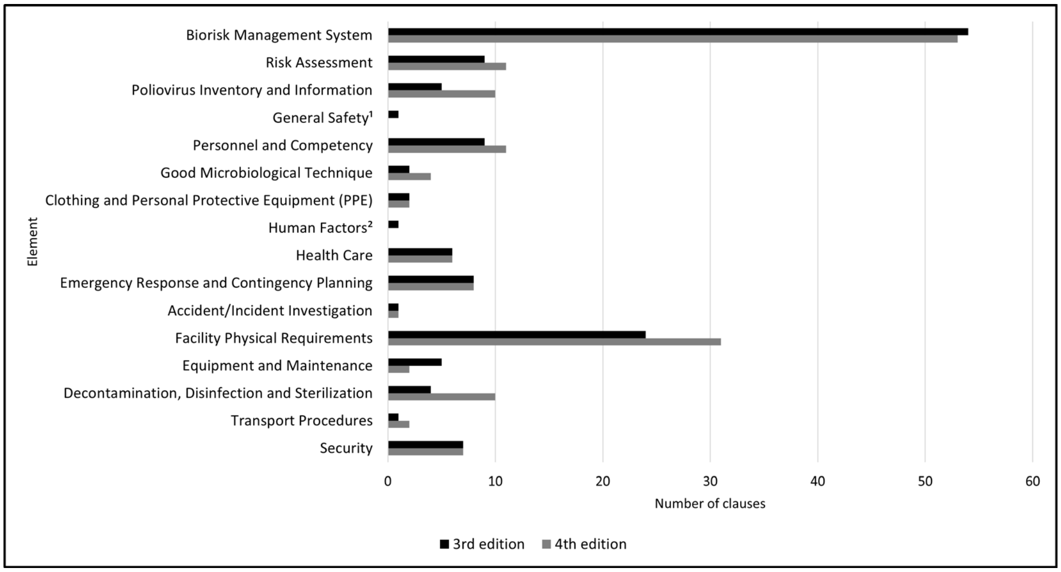

[1] Third edition clause for general safety was combined with risk assessment element in the fourth edition.

[2] Third edition clause for human factors was combined with personnel and competency element in the fourth edition.

Supplemental Figure S1. Number of elements and clauses in the poliovirus containment standard by edition.

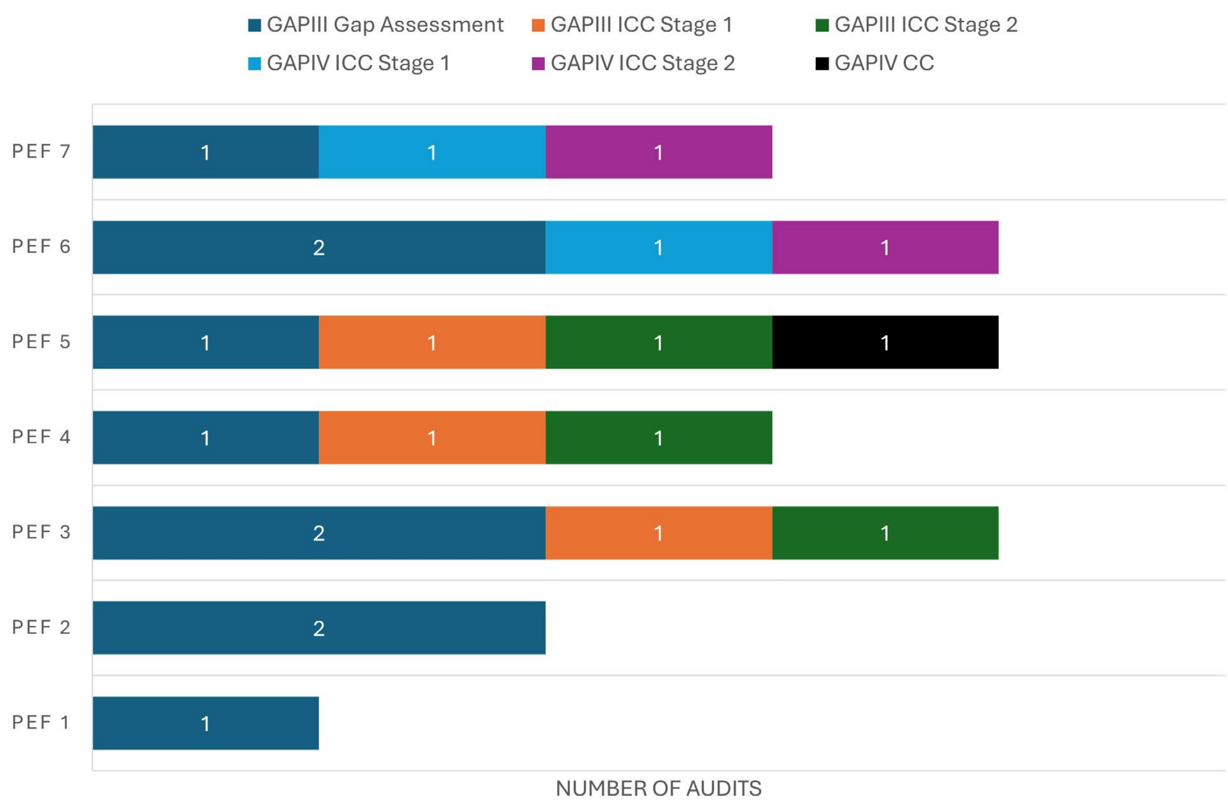

Supplemental Figure S2. U.S. NAC Poliovirus Containment Audits, 2017 – 2024. US NAC conducted 10 gap assessments using GAPIII at seven potential PEFs retaining PV2/WPV3/VDPV3 infectious materials, with assessments repeated at PEFs 2, 3, 6. After gap assessments, PEFs 1 and 2 destroyed poliovirus containment strains and did not apply for an ICC. U.S. NAC conducted six ICC audits at PEFs 3, 4, 5 using GAPIII, a total of 16 GAPIII audits. In 2024, US NAC conducted four ICC audits at PEFs 6, 7 and one CC audit at PEF 5 using GAPIV. GAP – Global action plan; ICC – Interim Certificate of Containment; CC – Certificate of Containment.
